# Evaluation of an electrostatic particle ionization technology for decreasing airborne pathogens in pigs

**DOI:** 10.1007/s10453-015-9413-3

**Published:** 2015-12-08

**Authors:** Carmen Alonso, Peter C. Raynor, Peter R. Davies, Robert B. Morrison, Montserrat Torremorell

**Affiliations:** 1 Department of Veterinary Population Medicine, College of Veterinary Medicine, University of Minnesota, 385 ASVM, 1988 Fitch Ave, Saint Paul, MN 55108 USA; 2 Division of Environmental Health Sciences, School of Public Health, University of Minnesota, Saint Paul, MN USA

**Keywords:** Influenza virus, *Staphylococcus aureus*, Porcine reproductive and respiratory syndrome virus, Porcine epidemic diarrhea virus, Aerosols, Electrostatic particle ionization

## Abstract

Influenza A virus (IAV), porcine reproductive and respiratory syndrome virus (PRRSV), porcine epidemic diarrhea virus (PEDV) and *Staphylococcus aureus* are important swine pathogens capable of being transmitted via aerosols. The electrostatic particle ionization system (EPI) consists of a conductive line that emits negative ions that charge particles electrically resulting in the settling of airborne particles onto surfaces and potentially decreasing the risk of pathogen dissemination. The objectives of this study were to determine the effect of the EPI system on the quantity and viability of IAV, PRRSV, PEDV and *S. aureus* in experimentally generated aerosols and in aerosols generated by infected animals. Efficiency at removing airborne particles was evaluated as a function of particle size (ranging from 0.4 to 10 µm), distance from the source of ions (1, 2 and 3 m) and relative air humidity (RH 30 vs. 70 %). Aerosols were sampled with the EPI system “off” and “on.” Removal efficiency was significantly greater for all pathogens when the EPI line was the closest to the source of aerosols. There was a greater reduction for larger particles ranging between 3.3 and 9 µm, which varied by pathogen. Overall airborne pathogen reduction ranged between 0.5 and 1.9 logs. Viable pathogens were detected with the EPI system “on,” but there was a trend to reducing the quantity of viable PRRSV and IAV. There was not a significant effect on the pathogens removal efficiency based on the RH conditions tested. In summary, distance to the source of ions, type of pathogen and particle size influenced the removal efficiency of the EPI system. The reduction in infectious agents in the air by the EPI technology could potentially decrease the microbial exposure for pigs and people in confinement livestock facilities.

**Electronic supplementary material:**

The online version of this article (doi:10.1007/s10453-015-9413-3) contains supplementary material, which is available to authorized users.

## Introduction

Among all infectious agents affecting swine, airborne pathogens are the most costly and difficult to control (Hyslop 1971). Porcine reproductive respiratory syndrome (PRRS), influenza A (IA), foot and mouth disease (FMD), classical swine fever (CSF) and porcine epidemic diarrhea (PED) viruses are important swine pathogens that spread via aerosols (Stärk 1999). All these pathogens are responsible for causing devastating losses in pig farms, specifically those located in high swine dense regions, due to their ability to spread rapidly and, in some instances, cause zoonotic infections. Unfortunately, there are limited options to limit the spread of airborne pathogens.

Viruses and bacteria that become airborne travel as part of particulate matter (PM) of various origins and sizes (Dutkiewicz et al. 1994). PM from livestock houses includes indoor airborne pollutants (viable and non-viable) that may be detrimental for animal performance and the health and well-being of animals and farmers (Donham and Leininger 1984; Donham 1991; Spencer et al. 2004). PM is considered a health hazard due to irritant effects on the respiratory tract, increased susceptibility to respiratory diseases and its role as a vehicle of transmission of viruses and bacteria from livestock (Harry 1978). PM concentration and size distribution depend on factors related to animal housing and feeding, animal type, season and sampling period within a day (Ellen et al. 2000).

A first step in decreasing infectious aerosol concentrations is PM reduction. Proposed strategies to reduce PM in livestock systems included the use of “low-dust” feed and feeding techniques (Pedersen et al. 2000; Takai and Pedersen 2000), use of feed additives (Takai et al. 1996), water or oil sprinkling (Nonnenmann et al. 2004; Senthilselvan et al. 1997; Takai and Pedersen 2000), changes in ventilation rates and air distribution (Aarnink and Wagemans 1997), and electrostatic precipitation and ionization (Cambra Lopez et al. 2009; Mitchell and King 1994; Rosentrater 2003; Stgeorge and Feddes 1995; Yao et al. 2009). Air washers equipped with UV irradiation systems have been used to specifically kill bacteria and viruses in bioaerosols, but are not commonly used in livestock production due to their limited capacity for handling large volumes of air in livestock buildings (Schulz et al. 2013).

The concept of utilizing ionization as a means to reduce or eliminate airborne particles or microbial levels has been reported previously. The use of ion emissions combined with photocatalytic oxidation demonstrated significant pathogen removal efficiency and biocidal capabilities (Grinshpun et al. 2007). Ionization has been tested in poultry houses and hatching cabinets (Mitchell et al. 2000, 2002; Richardson et al. 2002), pigs (Rosentrater 2003), cattle (Dolejs et al. 2006) and rabbits (Chiumenti and Guercini 1990). Ionization is considered more efficient in removing particles from the air than conventional techniques such as water or oil sprinkling, changes in ventilation rates or changes in air distribution (Daniels 2001). An electrostatic particle ionization (EPI) technology, with agricultural application in livestock, became commercially available recently. This technology consists of a long ionizer bar with sharp point electrodes connected to a power supply of high voltage (−30 kV) that generates a high negative ion output which charges airborne particles electrically. The ionized airborne particles are attracted toward opposite charges and in an enclosed space, like a confinement rearing facility, may be cleared from the air by adhesion to the walls or other charged surfaces (Mitchell 1997).

Ionization systems for use in swine facilities have not yet been fully developed despite preliminary research indicative of improved air quality (Rosentrater 2003). Ion density (related to the distance to the ion source and the number of sources), size of particles, PM concentrations, ventilation rates and humidity levels are factors that influence the performance of ionization in livestock facilities (Cambra-Lopez et al. 2010). More research is needed to evaluate the effectiveness of ionization to reduce particles, pathogen load and viability of infectious agents important to livestock. This is particularly relevant for food animals raised in confinement conditions such as pigs and poultry where animals are housed in enclosed environments and, specifically for pigs where the importance of airborne transmission for swine and zoonotic pathogens such as porcine reproductive and respiratory syndrome virus (PRRSV), porcine epidemic diarrhea virus (PEDV), influenza A virus (IAV) and methicillin-resistant *Staphylococcus aureus* (MRSA) is well documented. For zoonotic agents such as influenza, airborne spread from animals to people is of great concern as pigs can serve as “mixing vessels” that generate new viruses (Ito 2000), and large populations of people can be put at risk very quickly, as occurred during 2013 with H3N2 variant influenza infections associated with agricultural fairs (Bowman et al. 2014; Wong et al. 2012). In addition, *S. aureus* is the most common bacteria readily found in the air of swine barns, and personnel working in swine facilities is frequently colonized with *S. aureus* in their noses (Denis et al. 2009; Garcia Graells et al. 2012; Van Cleef et al. 2010), emphasizing the need to develop strategies to inactivate infectious aerosols.

The objectives of this study were to evaluate the efficiency of the EPI technology at reducing the quantity and viability of IAV, PRRSV, PEDV and *S. aureus* in mechanically generated aerosols (Study 1) and in aerosols emitted by infected animals (Study 2). We evaluated the impact of size of the airborne particles, relative humidity (RH) levels and distance to the source of ions on removal efficiency. Understanding the capabilities of this technology as a potential strategy for mitigating airborne pathogen spread is important for assessing the opportunity to improve both animal and human health.

## Materials and methods

### System design and testing protocol

All procedures were approved by the University of Minnesota Institutional Animal Care and Use Committee (IACUC) and Institutional Biosafety Committee (IBC).

Studies were primarily performed in the BSL-2 research animal units at the University of Minnesota campus in St. Paul. The BSL-2 animal unit was mechanically ventilated with negative pressure of 0.11 in. of water, included incoming and outgoing filtered air, and had a total air space of 35.1 m^3^. Environmental conditions of RH (%) and temperature (°C) were monitored continually.

For the specific aim of evaluating the effect of RH, an environmentally controlled chamber located in the Department of Mechanical Engineering at the University of Minnesota Minneapolis campus was utilized (Ge 2014). The chamber measured 1.95 m wide by 1.95 m deep by 1.45 m tall and was equipped with clear acrylic door and hypalon gloves to safely manipulate and retrieve instruments. To control the temperature inside the chamber, the walls and floor were constructed using plate heat exchangers attached to aluminum plates. A water heater and a water chiller were connected to adjust the temperature of the heat exchanger fluid. The chamber was insulated and HEPA-filtered, and air flow, negative pressure, temperature and RH were monitored and recorded at all times.

All variables and factors are described in Table 1. For Study 1, the total number of tests was 27 (3 pathogens × 3 heights of the EPI line × 3 replicates = 27). For the naturally generated particles or Study 2, the total number of tests was 40 [(17 days × 2 times) + (6 days × 1 time) = 40].

**Table 1:** Summary of study parameters

Study parameters	Study 1	Study 2
Aerosol source	Mechanically generated (6-jet Collison nebulizer)	Naturally generated (infection in pigs)
Aerosol generation height	2.8 m^a^	0.3 m
Pathogens tested	IAV, PRRSV and *S. aureus*	IAV, PRRSV and PEDV
EPI line height	3, 2, 1 m	1.3 m
Air collector locations	0.2 m	1.2 m
Optical particle counter location	0.2 m	1.2 m
Ionizer performance analyzer location	0 m	0 m
Replicates	3	2 (17 days) or 1 (6 days)
Total tests run	27	40

^a^All height distances measured above floor

The methods for each of the studies are described in the following sections.

### Aerosol generation

#### Study 1: Experimental pathogen aerosolization

Suspensions of 10^6^ and 10^5^ tissue culture infective dose per mL (TCID50/mL) of IAV A/Swine/Iowa/00239/2004 H1N1 and PRRSV strain MN-1-8-4, respectively, were suspended in phosphate-buffered saline (PBS) and used to generate aerosols. Both viruses, provided by the University of Minnesota Veterinary Diagnostic Laboratory, are known to be shed in aerosols by infected pigs (Cho et al. 2007; Dee et al. 2009; Corzo et al. 2014). For *S. aureus*, a 10^8^ colony-forming unit (CFU)/mL suspension of a methicillin-susceptible strain (MLST 398; spa type t034) isolated from a commercial pig was used.

Aerosols were generated continuously at 20 psi using a 6-jet Collison nebulizer (CN60, BGI, Inc, Waltham, MA) at a constant rate for a total of 60 min per test. The nebulizer was located on a wooden platform attached to the north wall of the room, beside the air inlet, 2.8 m from the floor. Aerosols were produced at a rate of 1.1 mL/min and, according to the nebulizer manufacturer specifications, created aerosol particles with a mass median aerodynamic diameter of 1–3 μm (Thomas et al. 2008).

#### Study 2: Experimental virus infection in pigs

A group of 12 5-week-old pigs were purchased from an IAV, PRRSV, PEDV and *Mycoplasmahyopneumoniae* negative herd based on routine serologic and antigenic testing and clinical herd history. Ten pigs out of 12 were sedated using an intramuscular injection of Telazol^®^ (Fort Dodge Animal Health, Fort Dodge, IA, USA) at the recommended dose of 6 mg/kg and inoculated with PRRSV strain 1-8-4 and influenza A/Swine/Iowa/00239/2004 H1N1at 48 h post-arrival. Pigs were inoculated intranasally and intratracheally (IAV), and intramuscularly and intranasally (PRRSV) with 2 mL inoculum of each pathogen. Two of the pigs were removed from the room prior to inoculation and commingled back with the rest of the pigs at 6 h after inoculation to serve as contact controls. At day 21 of the study, all pigs were intragastrically inoculated with a 20 mL solution of PEDV-positive material obtained from PEDV-infected pigs. The inoculation material was confirmed positive by PEDV RT-PCR and diluted to a cycle threshold (Ct) value of 15–16. The inoculation material was prepared and kept refrigerated for 24 h at 4 °C prior to inoculation.

### Electrostatic particle ionization (EPI) system

The electrostatic particle ionization system (EPI air, Baumgartner Environics, Inc, Olivia, MN, USA), consisting of stainless steel corona points attached to a stainless steel cable, was installed along the length of the animal isolation room. Corona points were suspended by a wooden frame and energized with 30,000 V of electricity at a low current of 2 mA by a specially designed corrosion-resistant power supply. The power supply was mounted inside the room and operated at 110 V and 60 Hz.

Specifically for Study 1, the EPI line was located at heights of 1, 2 and 3 m from the floor (online resource 1). During each test, the system operated in “off” position for 30 min for the collection of the first sample; then, it was turned “on” for 15 min for the aerosols to reach steady state. It then remained “on” for another 30 min for the next air sample collection. The time to reach steady state with the system “off” and “on” in mechanically generated aerosols had been determined in a set of previous studies (unpublished data).

During Study 2, the EPI line was placed at a height of 1.3 m from the floor (online resource 2). During each test, the system operated in “off” position for 60 min as air samples were taken; then was turned “on” for 15 min; and then remained “on” for another 60 min for additional air sampling.

### Sampling procedures

#### Pathogen sampling

Aerosols were collected using a liquid cyclonic collector (Midwest Micro-Tek, Brookings, SD, USA) (Corzo et al. 2013), an Anderson cascade impactor (ACI) (Thermo Electron Corporation, Waltham, MA, USA) (Appert et al. 2011), as well as a viable ACI. The two latter collectors separate aerosolized virus or bacteria by associated particle sizes. For Study 1, the air samplers were located 0.2 m above from the floor and kept 0.9 m apart (online resource 1). For Study 2, air samples were collected twice daily (9 a.m. and 3 p.m.) for 17 days and once (9 a.m.) for 6 days. Samplers were hung 1.2 m from the floor and were also 0.9 m apart (online resource 2). The pigs did not have direct contact or access to the devices.

Sample collection, using the cyclonic air collector, which draws air at 200 L/min, was carried out for 30 min. Collection media during sampling were 10 mL of PBS, or 10 mL of minimum essential media (MEM) supplemented with 4 % of bovine serum albumin for the mechanically generated aerosols (Study 1) and the aerosols generated by infected animals (Study 2), respectively. After collection, an average of 4 mL of sample was recovered, divided into 2 aliquots and stored at −80 °C. The collector was then disinfected with 70 % ethanol, rinsed with distilled water and dried with paper towels. After disinfection, the collection vessel and the turbine were swabbed to serve as controls, and samples were processed and stored in sterile plastic tubes at −80 °C until analysis.

The ACI samples air at 28.3 L/min and separates particles into eight stages with particle size diameter cut points of 0.4, 0.7, 1.1, 2.1, 3.3, 4.7, 5.6 and 9.0 µm. Air samples were collected for 30 min in Study 1 and 60 min in Study 2. After each sampling period, samples from the ACI were eluted from the plate on each stage using a cell scraper and 1 mL of PBS or MEM (Appert et al. 2011). All samples were transferred into 1.5 mL sterile plastic tubes, placed on ice and stored at −80 °C until testing.

Specifically for *S. aureus*, air samples were taken using a 6-stage viable ACI with Columbia-CNA agar plates able to capture bacteria associated with particles with size diameter cut points of 0.7, 1.1, 2.1, 3.3, 4.7 and 5.6 µm. After each sampling period, plates were removed, inverted in their covers, incubated for 12–18 h at 37 °C, and CFUs were counted as previously described (Andersen 1958; Butera et al. 1991). After each sampling, the ACI was disassembled and collection plates were scrubbed and disinfected with alkyl dimethyl benzyl ammonium chloride soap (Lysol, Reckitt Benckiser) and finally rinsed and dried with paper towels. After disinfection, a random number of collection plates and individual ACI stages were swabbed, and samples stored at −80 °C.

In order to monitor for infection status in pigs challenged in Study 2, oral fluids (Detmer et al. 2011; Romagosa et al. 2012), serum samples and fecal swabs were collected to test them by RT-PCR for IAV, PRRSV and PEDV as described below.

#### Total particle testing

Total airborne particle counts by particle size were collected using an optical particle counter (OPC) (AeroTrak 9306 Handheld Particle Sizer, TSI Inc., St. Paul, MN, USA) for 30 min at each sampling event of Study 2.

#### Ion decay testing

In order to ascertain the ion density released by the EPI system, ion decay readings were measured in triplicate at a location between both air samplers using an ionizer performance analyzer (Model 287, Monroe Electronics, Inc., NY, USA). The ionizer performance analyzer measures the time in seconds it takes for the positive ions accumulated on a voltage plate to decay from 1050 V to zero after interacting with the negative ions released in the room. The smaller the decay time, the faster the charge releases from the voltage plate, and the higher the ion concentration.

### Relative humidity test

In order to generate IAV aerosols, a 6-jet Collison nebulizer was utilized as described in methods earlier, and aerosols were sampled using an ACI for 15 min with the EPI system “off” and “on.” RH conditions were set at 30 and 70 % (±2 %), and three replicates were performed at each RH level.

### Diagnostic assays

Oral fluid samples, nasal swabs, serum samples and fecal swabs were tested for quantitative PRRSV, IAV and PEDV RT-PCRs as previously described (Alonso et al. 2014; Cho et al. 2006; Slomka et al. 2010). After incubation, *S. aureus* colonies on the agar plates were counted as previously described (Andersen 1958; Butera et al. 1991).

To assess the infectivity of the air samples collected using the air cyclonic collector, virus isolation was attempted from RT-PCR-positive samples in Madin–Darby canine kidney (MDCK) cells and a subline of the African monkey kidney cell line named MARC-145 cells for IAV and PRRSV, respectively. In the case of PEDV, a bioassay consisting of inoculating susceptible piglets with air samples testing RT-PCR positive was performed because of the difficulty to grow the virus in cell culture. Briefly, four 10-day-old pigs from a PEDV-negative farm were purchased and each pig allocated to a separate isolation room. On arrival, pigs were rectal swabbed and confirmed negative by PEDV RT-PCR. Inoculation material for each pig consisted of a pool of three air samples containing the collection media of the cyclonic air collector with the system “off” (two pigs) and another pool of three samples collected with the system “on” (one pig). The fourth pig was kept as a negative control. Each pig was intragastrically inoculated as previously described (Alonso et al. 2014) with 2 mL of the pooled air samples diluted 1:10 with PBS to obtain a total of 20 mL of inoculation material per pig. All pigs were euthanized 4 days post-inoculation by injection of 2 mL of pentobarbital (Fatal-Plus^®^, 100 mg/kg IV) into the external jugular vein.

### Statistical analysis

Data of RNA copies/m^3^ of air for PRRSV, IAV and PEDV, and number of *S. aureus* CFUs/m^3^, ion decay time, type of air sampler, replicate, distance of the EPI system to the ground and type of pathogen were consolidated in a spreadsheet (Microsoft EXCEL; Microsoft Corporation, Redmond, Washington, USA) and organized for analysis. Means, standard deviations, minimum and maximum values for quantitative variables, and frequency counts and percentages for categorical variables were calculated for descriptive analysis. For each of the studies, differences in the total pathogen concentrations in air and particle size-specific concentrations with the EPI system “on” and “off” were assessed for significance using PROC MIXED in SAS 9.3 (SAS Institute, Cary, North Carolina, USA) a mixed linear regression model for random and fixed effects. Results from all stages of the ACI were considered in the analysis when at least one of the eight stages that integrate the air sampler had a Ct value within the positive or suspect ranges. Negative results included in the analysis had a value of 100 RNA copies according to the limit of detection of the RT-PCR technique. Removal efficiency by the EPI system was calculated for each of the agents, and it was defined as the initial concentration of aerosolized virus or bacteria with the EPI system “off” minus final concentration with the EPI system “on” divided by initial concentration (Wu et al. 2006).

## Results

### Study 1: mechanically generated aerosols

#### Virus quantification in air samples

A total of 144 ACI stages and 18 cyclonic collector air samples were analyzed by IAV and PRRSV RT-PCR with the system “off” and “on.” Viral suspensions in the nebulizer had a mean RNA copies/mL of 1.53 × 10^8^ for IAV and 1.41 × 10^9^ for PRRSV.

The estimated mean of IAV RNA copies/m^3^ of air measured by the ACI ranged from 1.33 × 10^2^ to 1.21 × 10^4^ with the EPI system “off” and from 0 to 3.0 × 10^2^ with the EPI system “on.” The measurements using the cyclonic air collector ranged from 6.7 × 10^3^ to 2.6 × 10^4^ with the EPI system “off” and from 7.8 × 10^3^ to 3.0 × 10^4^ with the system “on.”

The estimated mean of PRRSV RNA copies/m^3^ of air measured by the ACI ranged from 8.30 × 10^2^ to 2.48 × 10^6^ with the EPI system “off” and from 0 to 4.98 × 10^5^ with the EPI system “on.” The values using the cyclonic collector ranged from 3.0 × 10^4^ to 7.3 × 10^5^ with the EPI system “off” and from 3.9 × 10^4^ to 2.8 × 10^5^ with the system “on.” For both viruses, negative controls tested negative.

The effect of the EPI system on the quantity of IAV by particle size is shown in online resource 3. In Study 1, IAV was found in all particle size ranges with the system “off” and in most of the size ranges with the system “on” except for particle sizes of 5.8–10 µm at 2 m distance and for particles of 4.7–5.8 and >9.0 µm at 3 m distance. There was a reduction in the number of RNA copies/m^3^ with the system “on,” and this reduction was greater at 3 m distance of the EPI line to the ground. Results from the reduction difference predicted by the model indicated a total reduction between 0.56 logs (for particles between 2.1 and 3.3 µm) and 2.58 logs (for particles between 3.3 and 4.7 µm) at 3 m distance of the EPI line to the ground.

The effect of the EPI system on the quantity of PRRSV detected by particle size is shown in online resource 4. PRRSV was found in all particle size ranges with the system “off” and in most of the sizes with the system “on,” except for the largest particle of >9.0 µm at 2 m distance. There was an overall reduction in the number of RNA copies/m^3^ with the system “on,” and this reduction was greater and significant for virus associated with larger particles (3.3–10.0 µm) at 3 m distance of the EPI line to the ground. The results indicated a total reduction between 1 log (for particles between 0.4 and 2.1 µm) and 3.8 logs (for particles between 4.7 and 5.8 µm) at 3 m distance of the EPI line to the ground.

#### Bacterial quantification in air samples

A total of 108 plates collected using the viable ACI were incubated and analyzed. The estimated mean of *S. aureus* CFUs/m^3^ of air measured by the ACI ranged between 1.88 × 10^1^ and 2.87 × 10^3^ with the EPI system “off” and from 0 to 1.43 × 10^3^ with the system “on.” Plates incubated with the air samples collected with the cyclonic collector had too many CFUs to count, and results are not reported. Negative controls tested negative.

The effect of the EPI system on the quantity of *S. aureus* detected by particle size is shown in online resource 5. *S. aureus* was found in all particle size ranges with both the system “off” and “on.” There was a reduction in the number of CFUs/m^3^ with the system “on,” and this reduction was greater at 3 m distance of the EPI line to the ground. These results indicated a total predicted reduction difference of 0.62 logs (for particles between 0.7 and 1.1 µm) to 1.35 logs (for particles between 4.7 and 5.8 µm) at 3 m distance of the EPI line to the ground.

#### Virus and bacteria removal efficiency

**Fig. 1:**
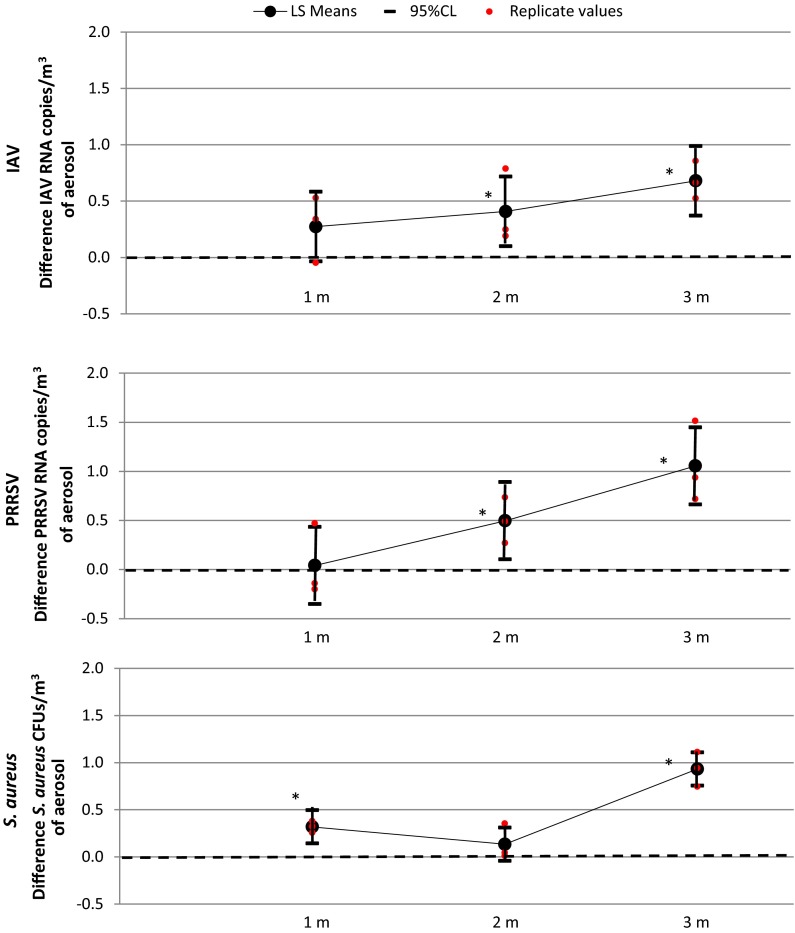
Linear regression modeling for total reduction in influenza A virus (IAV), porcine reproductive and respiratory virus (PRRSV) and *Staphylococcus aureus* with the EPI system “on” at various distances from the ground. Least square means of concentration difference of RNA copies/m^3^ or CFUs/m^3^ of aerosol by distance of the EPI line to the ground with the Andersen cascade impactor sampler after adding all pathogen particles of all stages. *Dashed line* indicates the null value. *95 % CI does not include the null value, *p* value <0.05

Removal efficiency was greater for all pathogens when the EPI line was located at 3 m from the ground and closer to the source of aerosols. Removal efficiency measured at 3, 2 and 1 m from the floor varied from 87.6 to 99, 22.4 to 65 and −10.7 to 51.7 % for IAV, PRRSV and *S. aureus,* respectively. The EPI system significantly reduced airborne IAV and PRRSV when the EPI line was located at 2 and 3 m from the floor, and at 1 and 3 m for aerosols containing *S. aureus* (Fig. 1).

#### Ion decay time

A total of 186 readings were taken during the study. Average ion decay time was 1.18 s (±0.02), 9.4 s (±0.76) and 44.18 s (±17.08) at 1, 2 and 3 m, respectively. Distance from the ion analyzer to the EPI lines, which is directly related to electrostatic field strength, played a statistically significant role after adjusting by replicate (*p* value <0.0001).

#### Relative humidity test

Results from the relative humidity performance test of the EPI system demonstrated a difference in the number of IAV RNA copies/m^3^ with the system “on” under the two different RH scenarios. A greater removal efficiency of 89 % was observed at 70 % RH compared to 47% at 30 % RH. However, this difference was not statistically significant (*p* value = 0.26).

### Study 2: aerosols released by infectious animals

#### Virus quantification in air samples

All pigs were confirmed positive by RT-PCR for IAV, PRRSV and PEDV after inoculation. All pigs tested positive for IAV in nasal swabs on day 3 (inoculated pigs) and on day 7 (contact pigs) after infection (RT-PCR Ct 24.6 ± 2.2 and 21.1 ± 2.8, respectively). All inoculated pigs experienced the peak of PRRSV viremia (1.25 × 10^9^ ± 8.93 × 10^8^ ORF6 RNA/mL) on day 7, and contact pigs experienced the peak on day 13 (4.57 × 10^8^ ± 2.96 × 10^8^ ORF6 RNA/mL).

**Fig. 2:**
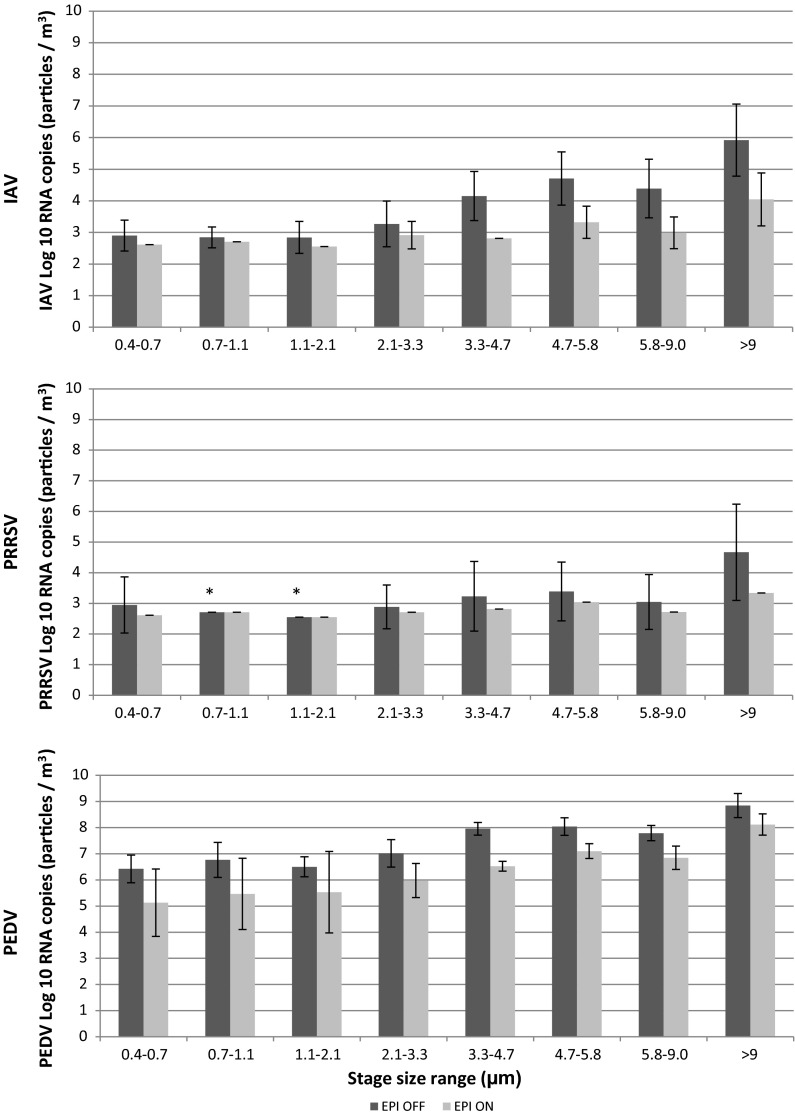
Concentration of influenza A virus (IAV), porcine reproductive and respiratory virus (PRRSV) and porcine epidemic diarrhea virus (PEDV) in the air (RNA copies/m^3^) after animal challenge with the EPI system “off” and “on” as a function of particle size. *Polymerase chain reaction (PCR) limit of detection

The total concentrations of IAV, PRRSV and PEDV associated with airborne particles of different sizes measured with the ACI with the EPI system “off” and “on” are shown in Fig. 2. Higher concentrations of viral particles were associated with larger particle sizes. There was a reduction in the number of viral particles/m^3^ for IAV, PRRSV and PEDV across all particle size intervals with the system “on.” However, in the case of PRRSV, RNA copies of the virus were not detected associated with particle sizes ranging from 0.7 to 2.1 µm with the system “off” or “on.”

**Fig. 3:**
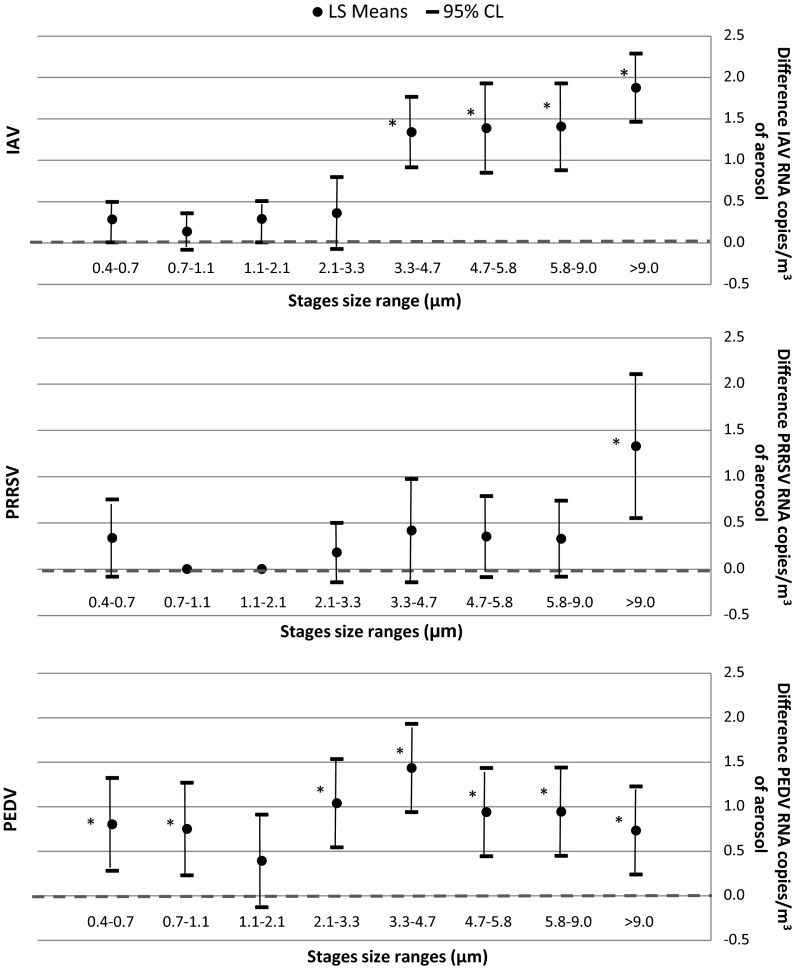
Linear regression modeling for total reduction in influenza A virus (IAV), porcine reproductive and respiratory virus (PRRSV) and porcine epidemic diarrhea virus (PEDV) from experimentally infected animals with the EPI system “on” by particle size. Least square means of concentration difference of virus per m^3^ of aerosol by particle size measured with the ACI sampler by different sizes. *Dashed line* indicates the null value. *95 % CI does not include the null value, *p* value <0.05

The predicted reduction difference per particle size between the system “off” and “on” obtained with the ACI collector was calculated for the three viruses (Fig. 3). The results indicated a total reduction from 0.14 logs (for particles between 0.7 and 1.1 µm) to a maximum of 1.90 logs (for particles >9 µm) for IAV, a reduction of 0.18 logs (for particles between 2.1 and 3.3 µm) to a maximum of 1.33 logs for the largest particles, and in the case of PEDV, we observed a reduction of 0.73 logs (for particles between 9.0 and 10.0 µm) to a maximum of 1.43 logs (for particles between 3.3 and 4.7 µm).

#### Total particle removal efficiency

Based on the optical particle counts obtained during Study 2, 82.8, 8.1, 2.3, 1.6, 2.0 and 3.2 % of total particles were distributed among the size ranges of 0.3–0.5, 0.5–1.0, 1.0–3.0, 3.5–5.0, 5.0–10 and larger than 10 µm, respectively. Removal efficiency for total particles ≥1 µm was greater than for those <1 µm and ranged between 76 and 82 % and between 52 and 56 % for these groups, respectively (Table 2). On the other hand, the removal efficiency of virus particles from aerosols generated by infectious animals ranged from 88 to 99.9 % for IAV, negative removal efficiency to 100 % for PRRSV and 58.8–96.8 % in the case of PEDV. Total particle size distribution measured by the OPC is shown in online resource 6.

**Table 2:** Removal efficiency by particle size of the EPI system for total particles and viral particles of influenza A virus (IAV), porcine reproductive and respiratory virus (PRRSV) and porcine epidemic diarrhea virus (PEDV) from aerosols generated by experimentally infected animals

Total particles (by optical particle counter)		Viral particles (by RT-PCR analysis)			
Particle size range (µm)	Average removal efficiency (%)	Particle size range (µm)	Average removal efficiency (%)		
			IAV	PRRSV	PEDV
0.3–0.5	51.6	0.4–0.7	77.1	98.4	89.9
0.5–1.0	56.1	0.7–1.1	52	–^a^	95.5
1.0–3.0	76.2	1.1–2.1	77.7	–	58.8
3.0–5.0	82.2	2.1–3.3	77.5	97.8	89.8
5.0–10.0	79.8	3.3–4.7	98.2	99.7	96.4
>10	76.2	4.7–5.8	96.5	98.8	89.7
		5.8–9.0	97	98.2	85.1
		>9.0	98.2	99.9	84.3

Removal efficiency (%) was calculated as the initial concentration of particles with the EPI system “off” minus final concentration of particles with the EPI system “on” divided by initial concentration of particles with the EPI system “off”

^a^Removal efficiency could not be calculated because both values obtained with the system “off” and “on” were the PCR limit of detection

#### Virus viability results

Of all air samples collected using the air cyclonic collector, 90, 17.6 and 100 % tested positive for IAV, PRRSV and PEDV by RT-PCR, respectively (Table 3). IAV was isolated in 6 out of 27 (22.2 %) positive air samples. From those, 5 were isolated with the EPI system “off” and 1 with the EPI system “on.” In the case of PRRSV, it was isolated in 9 out 12 (75 %) samples of which 6 were isolated from the system “off” and 3 with the system “on.” Results from the bioassay performed in pigs demonstrated the presence of infectious PEDV in samples treated with the EPI system “on” and “off.” All three inoculated pigs experienced moderate to severe diarrhea, with fecal number of RNA copies/mL ranging from 3.96 × 10^10^ to 7.57 × 10^10^. Pigs had histopathological lesions of moderate to marked atrophic enteritis, while the pig from the negative control group showed no clinical signs, had normal intestinal histomorphology and tested negative by RT-PCR.

**Table 3:** Number of positive results of influenza A virus (IAV), porcine reproductive and respiratory virus (PRRSV) and porcine epidemic diarrhea virus (PEDV) by RT-PCR and virus isolation (VI) or bioassay in air samples from acutely infected animals

Virus	Diagnostic technique	EPI system		Total samples
		“off”	“on”	
IAV	PCR^a^	14/15 (93.3 %)	14/15 (93.3 %)	27/30 (90.0 %)
	VI^b^	5/14 (35.7 %)	5/14 (35.7 %)	6/27 (22.2 %)
PRRSV	PCR	8/34 (23.5 %)	8/34 (23.5 %)	12/6 (17.6 %)
	VI	6/8 (75.0 %)	6/8 (75.0 %)	9/12 (75.0 %)
PEDV	PCR	6/6 (100 %)	6/6 (100 %)	12/12 (100 %)
	Bioassay^c^	2/2 (100 %)	2/2 (100 %)	3/3 (100 %)

^a^RT-PCR results presented as number of positives of total samples tested (%)

^b^VI results presented as positive samples of total RT-PCR positive samples tested (%)

^c^Bioassay results are presented as positive pigs of the total inoculated (%)

## Discussion

Developing biocontainment strategies for production animals is a priority for food animal industries. A particular challenge is the prevention of spread of emergent pathogens of economic (i.e., PRRSV, PEDV) and zoonotic importance (i.e., IAV, *S. aureus)* than may be transmitted among farms via aerosols. Our results indicate an overall reduction on the quantity of airborne pathogens in air treated with the EPI system. However, distance to the source of ions, particle size and type of infectious agent affected the removal efficiency of the system. Notably, the EPI system produced a reduction in the number of viable airborne viruses within the airspace of infected pigs.

In translating this approach to industry, it is important to understand whether the physical orientation of the system will influence pathogen reduction. We found that removal efficiency was influenced by pathogen type and location of the EPI line. Distance to the ion source greatly influenced the efficiency of the system resulting in higher ion concentrations as distance to the EPI line decreased. Greater removal efficiency was observed for influenza and PRRS viruses than for *S. aureus*. Similar results have been found for *Bacillus subtilis* vegetative cells, *Pseudomonas fluorescens*, *Candida albicans*, toxins and allergens mechanically generated (MacFarlane et al. 1999; Xie et al. 2011; Yao et al. 2009). This is the first report showing that proximity to the source of ions also influences reduction in airborne viruses.

Removal efficiency of viruses by the EPI system was shown for mechanically generated aerosols and for aerosols generated by experimentally infected animals. In the case of IAV, our results demonstrated a difference in concentration between 0.35 and 1.9 logs after the EPI treatment on particles ranging between 2.1 and >9 µm containing viable virus. This log difference can be translated to a removal efficiency of 2.3 and 98 %, respectively. Considering that virus particles are positively associated with larger size ranges, and that previous studies did not report their results based on particle size, this total removal efficiency could be considered higher than that of the use of feed additives, changes in ventilation rates and air distribution, and other electrostatic precipitators (Takai et al. 1996; Aarnink and Wagemans 1997; Cambra Lopez et al. 2009) but similar to a total of 85 % obtained when a combined method of spraying an oil mixture controlled by an animal activity sensor was used (Takai and Pedersen 2000). Removal efficiencies by particle size were higher for aerosols generated by animals than mechanically, particularly for IAV. It is unclear why the differences existed but stability and nature of aerosols generated by animals could have played a role. Non-biological particles such as NaCl carry fewer electric charges compared to biological particles (Mainelis et al. 2001), so results obtained from mechanically generated aerosols could underestimate the system efficiency due to their dilution on PBS media. Location of source of aerosols with regard to the air sampler’s position within the room (2.8 m height in Study 1 vs. 30 cm from the animals in Study 2) could have also influenced the results.

Our methods also allowed us to quantify the removal efficiency of total airborne particles and compare those to the viral removal efficiency of particles of different sizes. We observed that removal efficiency increased with particle size as measured by the OPC and these results were very consistent during the entire study. Notably, similar results were observed with the linear regression modeling analysis for IAV and PRRSV. For both viruses, total viral reduction was highest for particles ranging between 3.3 and those >9 µm diameter from aerosols generated by infected animals. Interestingly, total reduction in PEDV particles was the highest when associated with particles between 3.3 and 4.7 µm. For the nature and scope of this study, it remains unknown whether removal mechanisms of particles such as gravity, impaction or interception played a significant role in the total removal efficiency of virus. More research is needed to corroborate these results and evaluate whether other mechanisms besides ionization may have played a role in the removal efficiency parameters.

Virus viability results indicated a trend toward reduced viability of airborne pathogens when the EPI system was turned “on”. In the case of PEDV, we did not rely on cell culture methods because PEDV is difficult to grow in vitro, but assessed viability with a bioassay study. Although with the bioassay we were only able to assess presence or absence of virus, it provided an estimate of PEDV viability. However, we do not know whether the EPI system decreased the overall amount of PEDV in the air. Nevertheless, samples collected with the EPI system “on” were still infectious.

Our study also suggests potential for reducing exposure of zoonotic pathogens to swine workers and veterinarians. Removal efficiency of *S. aureus* (in Study 1) and IAV (in Study 2) was significant for all particle sizes measured (0.4 to >9 µm, with the exception of those ranging between 0.7 and 1.1 µm) at 3 m distance to the source of ions and those >3.3 µm in aerosols generated by infected animals. These results indicate, for the first time, the size of particles with which these zoonotic pathogens associate once aerosolized and the size ranges at which the system was more effective at removing them from the air. Decreasing the quantity of these pathogens in the occupational environment could reduce pathogen transmission between animals and people although further research is needed to determine whether this reduction is sufficient to halt transmission of zoonotic agents from animals to people.

Throughout the study, we found instances where the removal efficiency was negative (meaning there were more particles in the air after the EPI system was “on”). These results were unexpected, and they may be due to the sensitivity of the PCR technique where small variations in RNA copies can result in significant differences affecting the removal efficiency ratio. Alternatively, there could have been changes in the experimental conditions during the time the EPI system was “on” or “off” that could have affected the results although this is unlikely since negative removal efficiencies were not observed when removal efficiency was calculated for total airborne particle concentrations.

Relative humidity has also been reported to impact the efficiency of ionization (MacFarlane et al. 1999). However, under the conditions of our study, our results did not show a statistically significant effect of the EPI in IAV removal efficiency at the two RH tested although there was a trend toward higher efficiency at higher humidity. The relationship between negative ion concentration (an indicator of particle removal efficiency) and air humidity levels is complicated and involves hydration and chemical reactions of negative air ions with water in air (Wu et al. 2006). Further studies are needed to fully establish the impact of RH on the EPI system efficiency taking into account interactions with other environmental factors.

In conclusion, ionization of airborne particles reduces the quantity of airborne pathogens. Decreasing the infectious pathogen load should mitigate the risk of occupational exposure and should help contain pathogen spread from farms and improve overall regional biosecurity in pigs.

## Electronic supplementary material

Below is the link to the electronic supplementary material.

Online resource 1 A diagram of the research unit utilized in Study 1, depicting the release of mechanically generated aerosols, the location of the EPI line, the ion release, and the placement of the Andersen cascade impactor and liquid cyclonic air collectors during the collection of air samples (TIFF 204 kb)

Online resource 2 A diagram of the research unit utilized in Study 2, depicting the animal space, the location of the EPI line, the ion release, and the placement of the Andersen cascade impactor and liquid cyclonic air collectors during the collection of air samples (TIFF 190 kb)

Online resource 3 Concentration of influenza A virus (IAV) with the EPI system “off” and “on,” reduction efficiency, and predicted total reduction as a function of particle size and distance of the EPI line to the ground measured by RT-PCR in the different stages of the Andersen Cascade Impactor (DOCX 17 kb)

Online resource 4 Concentration of porcine reproductive and respiratory syndrome virus (PRRSV) with the EPI system “off” and “on,” reduction efficiency, and predicted total reduction as a function of particle size and distance of the EPI line to the ground measured by RT-PCR in the different stages of the Andersen Cascade Impactor (DOCX 17 kb)

Online resource 5 Concentration of colonies of *Staphylococcus aureus* with the EPI system “off” and “on,” efficiency, and predicted total reduction as a function of particle size and distance of the EPI line to the ground measured by bacterial culture in the different stages of the viable Andersen Cascade Impactor (DOCX 16 kb)

Online resource 6 Distribution of total airborne particles (geometric mean of number of particles/m^3^ and 95 % confident interval) measured using an optical particle counter with the EPI system “off” and the system “on” (PDF 76 kb)
