# Supplementary figures and images for: Evaluation of an electrostatic particle ionization technology for decreasing airborne pathogens in pigs
Source: Aerobiologia (Bologna). 2015 Dec 8;32(3):405–19. doi: 10.1007/s10453-015-9413-3 (PMC4996881; doi:10.1007/s10453-015-9413-3)

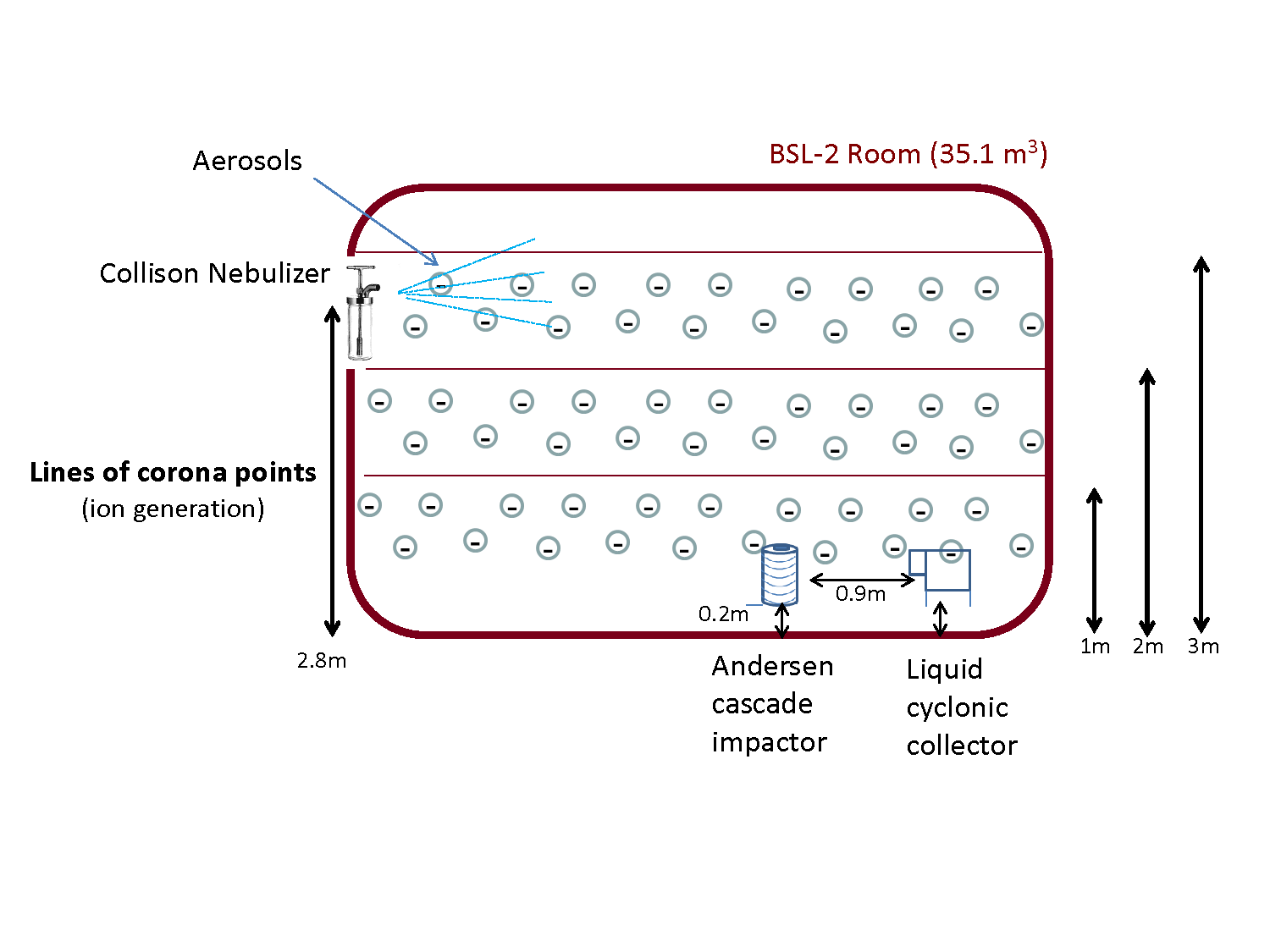

Supplement: Supplementary file 1 — A diagram of the research unit utilized in Study 1, depicting the release of mechanically generated aerosols, the location of the EPI line, the ion release, and the placement of the Andersen cascade impactor and liquid cyclonic air collectors during the collection of air samples (TIFF 204 kb) [file 10453_2015_9413_MOESM1_ESM.tiff]

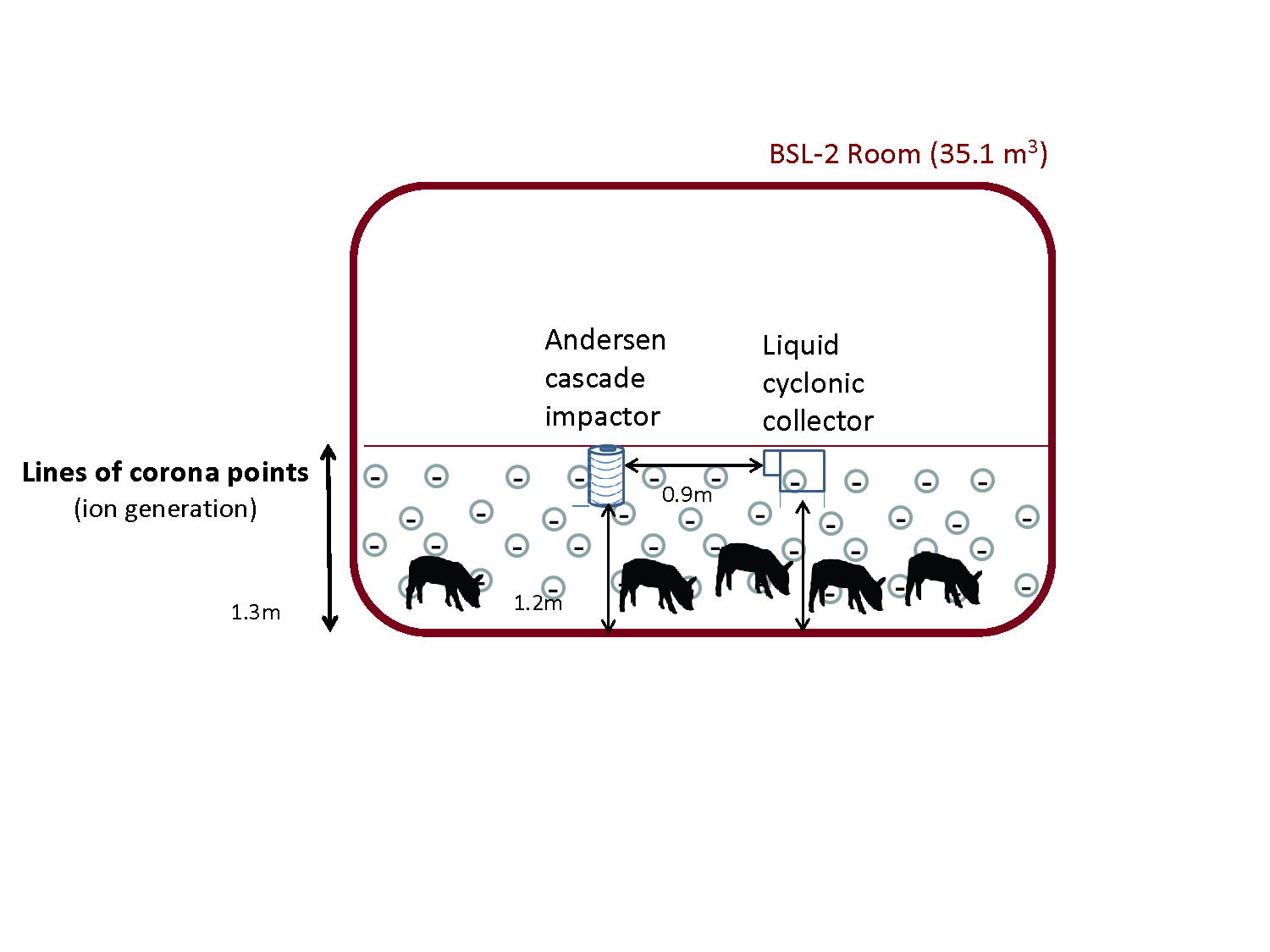

Supplement: Supplementary file 2 — A diagram of the research unit utilized in Study 2, depicting the animal space, the location of the EPI line, the ion release, and the placement of the Andersen cascade impactor and liquid cyclonic air collectors during the collection of air samples (TIFF 190 kb) [file 10453_2015_9413_MOESM2_ESM.tiff]

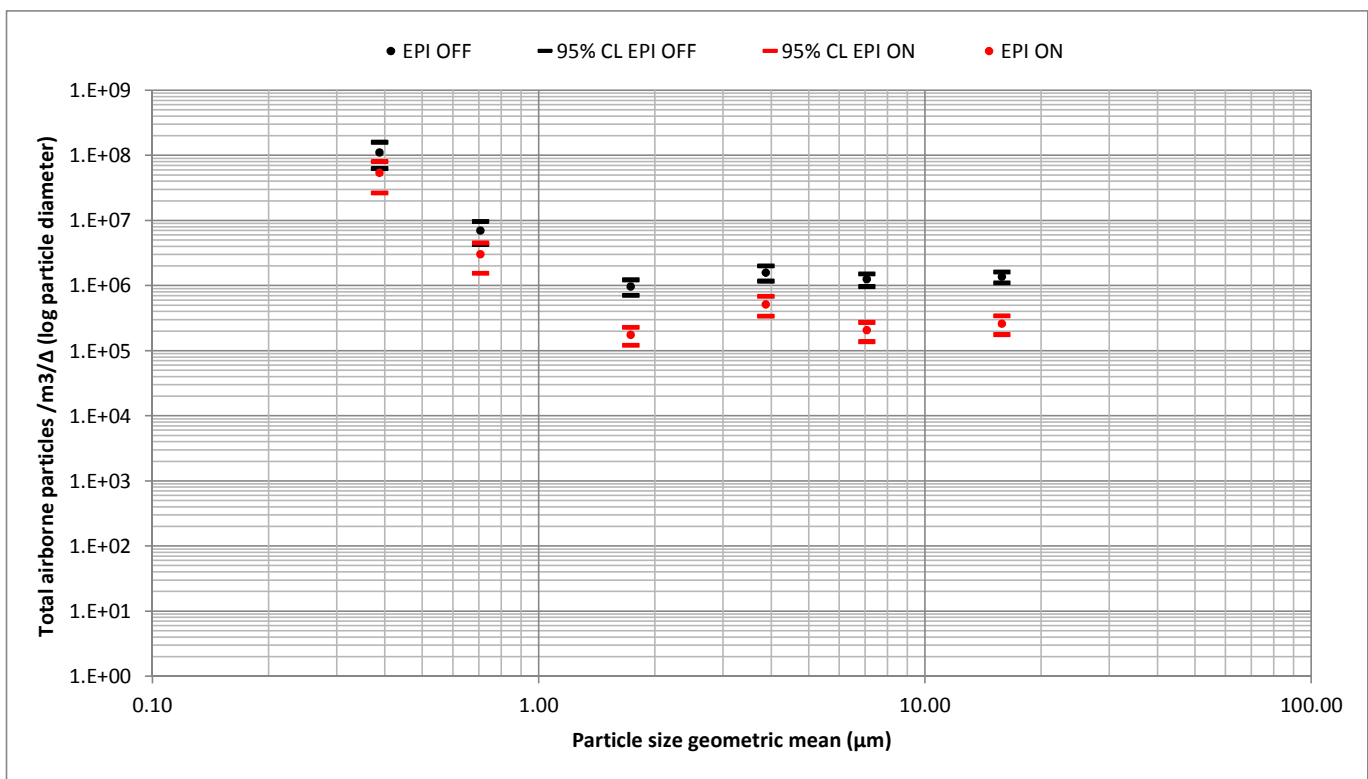

Supplement: Supplementary file 6 — Distribution of total airborne particles (geometric mean of number of particles/m3 and 95 % confident interval) measured using an optical particle counter with the EPI system “off” and the system “on” (PDF 76 kb) [file 10453_2015_9413_MOESM6_ESM.pdf]
