# Supplementary material for: Evaluation of an electrostatic particle ionization technology for decreasing airborne pathogens in pigs
Source: Aerobiologia (Bologna). 2015 Dec 8;32(3):405–19. doi: 10.1007/s10453-015-9413-3 (PMC4996881; doi:10.1007/s10453-015-9413-3)
Supplement: Supplementary file 3 — Concentration of influenza A virus (IAV) with the EPI system “off” and “on,” reduction efficiency, and predicted total reduction as a function of particle size and distance of the EPI line to the ground measured by RT-PCR in the different stages of the Andersen Cascade Impactor (DOCX 17 kb) [file 10453_2015_9413_MOESM3_ESM.docx]

Online resource 3. Concentration of influenza A virus (IAV) with the EPI system “off” and “on”, reduction efficiency, and predicted total reduction as a function of particle size and distance of the EPI line to the ground measured by RT-PCR in the different stages of the Andersen Cascade Impactor.

| EPI line level  (m) | Stage size range (µm) | Mean IAV concentration (RNA copies /m^3^)  EPI “off” EPI “on” | | Reduction  efficiency (%) | Predicted total reduction (log10 RNA copies/m^3^) | Predicted total reduction 95% CI |
| --- | --- | --- | --- | --- | --- | --- |
| 1 | 0.4-0.7 | 6.37E+03 | 1.48E+03 | 76.8 | 2.78 | (0.45, 5.10)* |
| 1 | 0.7-1.1 | 1.18E+04 | 3.40E+03 | 71.1 | 0.47 | (-1.85, 2.80) |
| 1 | 1.1-2.1 | 1.11E+04 | 5.52E+03 | 50.4 | 0.25 | (-2.08, 2.57) |
| 1 | 2.1-3.3 | 3.45E+03 | 4.74E+03 | -37.6 | -0.14 | (-2.46, 2.19) |
| 1 | 3.3-4.7 | 1.97E+03 | 3.11E+03 | -57.6 | -1.15 | (-3.48, 1.17) |
| 1 | 4.7-5.8 | 3.05E+03 | 1.40E+03 | 54.2 | 2.43 | (1.01, 4.75)* |
| 1 | 5.8-9.0 | 5.28E+02 | 3.00E+02 | 43.2 | 0.08 | (-2.43, 2.40) |
| 1 | > 9.0 | 9.27E+02 | 1.50E+03 | -61.7 | 0.95 | (-1.37, 3.28) |
| 2 | 0.4-0.7 | 3.90E+03 | 4.77E+03 | -22.1 | 1.09 | (-1.20, 3.41) |
| 2 | 0.7-1.1 | 6.53E+03 | 1.99E+03 | 69.5 | 1.58 | (-0.74, 3.90) |
| 2 | 1.1-2.1 | 9.52E+03 | 3.96E+03 | 58.4 | 1.58 | (-0.74, 3.91) |
| 2 | 2.1-3.3 | 5.10E+03 | 2.08E+03 | 59.1 | 1.45 | (-0.87, 3.78) |
| 2 | 3.3-4.7 | 1.81E+03 | 1.21E+03 | 33.3 | 1.20 | (-1.12, 3.53) |
| 2 | 4.7-5.8 | 8.95E+02 | 7.22E+02 | 19.3 | 0.03 | (-2.30, 2.35) |
| 2 | 5.8-9.0 | 1.01E+03 | 0.00E+00 | 100.0 | 1.26 | (-1.06, 3.59) |
| 2 | > 9.0 | 4.22E+02 | 0.00E+00 | 100.0 | 1.13 | (-1.19, 3.46) |
| 3 | 0.4-0.7 | 4.77E+03 | 1.95E+03 | 59.2 | 1.29 | (-1.03, 3.61) |
| 3 | 0.7-1.1 | 8.04E+03 | 2.17E+03 | 73.0 | 0.57 | (-1.75, 2.89) |
| 3 | 1.1-2.1 | 1.21E+04 | 9.82E+02 | 92.0 | 1.78 | (-0.54, 4.11) |
| 3 | 2.1-3.3 | 3.95E+03 | 1.29E+03 | 67.3 | 0.56 | (-1.76, 2.89) |
| 3 | 3.3-4.7 | 3.23E+03 | 6.23E+02 | 80.7 | 2.58 | (0.25, 4.91)* |
| 3 | 4.7-5.8 | 5.89E+02 | 0.00E+00 | 100.0 | 1.18 | (-1.14, 3.51) |
| 3 | 5.8-9.0 | 9.74E+02 | 1.04E+02 | 89.3 | 1.37 | (-0.95, 3.71) |
| 3 | >9.0 | 5.25E+02 | 0.00E+00 | 100.0 | 1.17 | (-1.16, 3.49) |

* If CI does not include null value, p value < 0.05
