# Supplementary material for: Evaluation of an electrostatic particle ionization technology for decreasing airborne pathogens in pigs
Source: Aerobiologia (Bologna). 2015 Dec 8;32(3):405–19. doi: 10.1007/s10453-015-9413-3 (PMC4996881; doi:10.1007/s10453-015-9413-3)
Supplement: Supplementary file 4 — Concentration of porcine reproductive and respiratory syndrome virus (PRRSV) with the EPI system “off” and “on,” reduction efficiency, and predicted total reduction as a function of particle size and distance of the EPI line to the ground measured by RT-PCR in the different stages of the Andersen Cascade Impactor (DOCX 17 kb) [file 10453_2015_9413_MOESM4_ESM.docx]

Online resource 4. Concentration of porcine reproductive and respiratory syndrome virus (PRRSV) with the EPI system “off” and “on”, reduction efficiency, and predicted total reduction as a function of particle size and distance of the EPI line to the ground measured by RT-PCR in the different stages of the Andersen Cascade Impactor.

| EPI line level  (m) | Stage size range (µm) | PRRSV concentration (RNA copies /m^3^)  EPI “off” EPI “on” | | Reduction  efficiency (%) | Predicted total reduction (log10 RNA copies/m^3^) | Predicted total reduction 95% CL |
| --- | --- | --- | --- | --- | --- | --- |
| 1 | 0.4-0.7 | 1.37E+04 | 4.36E+03 | 68.1 | 0.32 | (-1.33, 1.97) |
| 1 | 0.7-1.1 | 7.80E+04 | 5.65E+03 | 92.8 | 0.91 | (-0.74, 2.57) |
| 1 | 1.1-2.1 | 4.19E+04 | 5.50E+04 | -31.2 | -0.09 | (-1.73, 1.57) |
| 1 | 2.1-3.3 | 4.58E+04 | 4.49E+04 | 1.9 | -0.16 | (-1.81, 1.50) |
| 1 | 3.3-4.7 | 1.02E+04 | 2.18E+04 | -114.5 | -0.39 | (-2.04, 1.26) |
| 1 | 4.7-5.8 | 2.60E+03 | 8.67E+03 | -232.6 | -1.46 | (-3.11, 0.21) |
| 1 | 5.8-9.0 | 3.36E+03 | 1.05E+03 | 68.7 | 1.25 | (-0.40, 2.91) |
| 1 | > 9.0 | 8.30E+02 | 2.80E+02 | 66.3 | 1.14 | (-0.51, 2.81) |
| 2 | 0.4-0.7 | 2.01E+05 | 1.94E+04 | 90.3 | 1.08 | (-0.57, 2.72) |
| 2 | 0.7-1.1 | 3.50E+05 | 3.80E+04 | 89.1 | 0.98 | (-0.67, 2.63) |
| 2 | 1.1-2.1 | 2.33E+05 | 7.03E+04 | 69.8 | 0.50 | (-1.15, 2.15) |
| 2 | 2.1-3.3 | 8.25E+04 | 9.88E+04 | -19.7 | -0.06 | (-1.71, 1.59) |
| 2 | 3.3-4.7 | 4.38E+04 | 5.58E+04 | -27.4 | -0.05 | (-1.71, 1.60) |
| 2 | 4.7-5.8 | 2.42E+04 | 1.01E+04 | 58.2 | 0.38 | (-1.27, 2.04) |
| 2 | 5.8-9.0 | 2.48E+04 | 1.47E+03 | 94.1 | 3.37 | (1.71, 5.02)* |
| 2 | > 9.0 | 7.72E+03 | 0.00E+00 | 100.0 | 4.11 | (2.46, 5.77)* |
| 3 | 0.4-0.7 | 5.78E+05 | 4.29E+04 | 92.6 | 1.09 | (-0.58, 2.75) |
| 3 | 0.7-1.1 | 1.01E+06 | 8.84E+04 | 91.3 | 1.07 | (-0.58, 2.73) |
| 3 | 1.1-2.1 | 2.48E+06 | 4.98E+05 | 80.0 | 1.07 | (-0.59, 2.72) |
| 3 | 2.1-3.3 | 6.17E+05 | 2.83E+04 | 95.4 | 1.35 | (-0.31, 3.00) |
| 3 | 3.3-4.7 | 2.58E+05 | 1.46E+04 | 94.3 | 2.58 | (0.93, 4.23)* |
| 3 | 4.7-5.8 | 6.66E+04 | 1.13E+03 | 98.3 | 3.80 | (2.15, 5.45)* |
| 3 | 5.8-9.0 | 7.23E+04 | 2.54E+03 | 96.5 | 3.73 | (2.07, 5.38)* |
| 3 | >9.0 | 3.12E+04 | 2.08E+04 | 33.4 | 2.97 | (1.31, 4.62)* |

* If CI does not include null value, p value < 0.05
