# Supplementary material for: Evaluation of an electrostatic particle ionization technology for decreasing airborne pathogens in pigs
Source: Aerobiologia (Bologna). 2015 Dec 8;32(3):405–19. doi: 10.1007/s10453-015-9413-3 (PMC4996881; doi:10.1007/s10453-015-9413-3)
Supplement: Supplementary file 5 — Concentration of colonies of Staphylococcus aureus with the EPI system “off” and “on,” efficiency, and predicted total reduction as a function of particle size and distance of the EPI line to the ground measured by bacterial culture in the different stages of the viable Andersen Cascade Impactor (DOCX 16 kb) [file 10453_2015_9413_MOESM5_ESM.docx]

Online resource 5. Concentration of colonies of *Staphylococcus aureus* with the EPI system “off” and “on”, reduction efficiency, and predicted total reduction as a function of particle size and distance of the EPI line to the ground measured by bacterial culture in the different stages of the viable Andersen Cascade Impactor.

| EPI line level  (m) | Stage size range (µm) | *S. aureus* concentration (CFUs /m^3^)  EPI “off” EPI “on” | | Reduction  efficiency (%) | Predicted total reduction (log10 RNA copies/m^3^) | Predicted total reduction 95% CI |
| --- | --- | --- | --- | --- | --- | --- |
| 1 | 0.7-1.1 | 655 | 199 | 69.7 | 0.50 | (0.08, 0.93)* |
| 1 | 1.1-2.1 | 1,296 | 739 | 43.0 | 0.26 | (-0.16, 0.68) |
| 1 | 2.1-3.3 | 759 | 374 | 50.7 | 0.22 | (-0.21, 0.64) |
| 1 | 3.3-4.7 | 163 | 57 | 65.4 | 0.50 | (0.08, 0.92)* |
| 1 | 4.7-5.8 | 32 | 44 | -36.6 | 0.05 | (-0.37, 0.47) |
| 1 | 5.8-9.0 | 23 | 6 | 72.4 | 0.62 | (0.19, 1.04)* |
| 2 | 0.7-1.1 | 290 | 97 | 66.4 | 0.46 | (0.04, 0.74)* |
| 2 | 1.1-2.1 | 2,342 | 1,168 | 50.1 | 0.32 | (-0.11, 0.74) |
| 2 | 2.1-3.3 | 776 | 1,231 | -58.7 | -0.18 | (-0.6, 0.25) |
| 2 | 3.3-4.7 | 220 | 219 | 0.4 | 0.05 | (-0.38, 0.47) |
| 2 | 4.7-5.8 | 65 | 84 | -29.0 | -0.05 | (-0.47, 0.37) |
| 2 | 5.8-9.0 | 42 | 30 | 28.3 | 0.29 | (-0.13, 0.71) |
| 3 | 0.7-1.1 | 191 | 46 | 75.7 | 0.62 | (0.19, 1.04)* |
| 3 | 1.1-2.1 | 2,298 | 321 | 86.0 | 0.87 | (0.45, 1.29)* |
| 3 | 2.1-3.3 | 1,553 | 232 | 85.1 | 0.91 | (0.49, 1.34)* |
| 3 | 3.3-4.7 | 1,201 | 71 | 94.1 | 1.24 | (0.82, 1.67)* |
| 3 | 4.7-5.8 | 226 | 10 | 95.5 | 1.35 | (0.93, 1.78)* |
| 3 | 5.8-9.0 | 119 | 11 | 90.8 | 1.28 | (0.86, 1.71)* |

* If CI does not include null value, p value < 0.05
